# General anesthesia in patients with hepatic encephalopathy and acute variceal bleeding undergoing endoscopic treatment: A retrospective study

**DOI:** 10.1097/MD.0000000000034395

**Published:** 2023-08-25

**Authors:** Tao Chen, Lin Wen, Rui Zhong, Xia Chen

**Affiliations:** a Department of Digestive Endoscopy Center, Digestive Disease Center, Suining Central Hospital, Suining, China; b Department of Respiratory and Critical Care Medicine, Jiangyou Second People’s Hospital, Jiangyou, China; c Department of Gastroenterology, Clinical Medical College and The First Affiliated Hospital of Chengdu Medical College, Chengdu, China.

**Keywords:** acute variceal bleeding, endoscopic treatment, general anesthesia, hepatic encephalopathy

## Abstract

The management of cirrhotic patient with encephalopathy and acute variceal bleeding (AVB) remains a clinical challenge with a high mortality. Early endoscopic therapies are frequently applied in patients with AVB. However, the application of general anesthesia in endoscopic surgery for patients with hepatic encephalopathy (HE) is pretty challenging. The present study aimed to evaluate the possible effect of general anesthesia with tracheal intubation on patient complicated with encephalopathy and AVB during endoscopic procedure. Thirty-six cirrhotic patients with encephalopathy and AVB were retrospectively studied, 14 patients underwent endoscopic treatment under general anesthesia with tracheal intubation, and 22 patients received pharmacological treatment, or and endoscopic therapy without general anesthesia served as the control group. Routine clinical and laboratory data were collected. The total mortality rate was 13.9% (5/36), 2 (14.3%) in the anesthesia group, 3 (13.6%) in the control group. The child-Pugh class of death cases were all grade C, 3 (60%) of them were in the stage IV of HE. The stage of HE was all improved in the 2 groups, 12 (85.8 %) patients were totally recovered from consciousness disturbance in the anesthesia group and 16 (72.7 %) in the control group respectively, the difference between the 2 groups was not significant (*P*>0.05). Except the death cases, there were still 3 patients in the control group had impaired consciousness at discharge. Child-Pugh score, Child-Pugh class and the stage of HE in the anesthesia group were significantly improved at discharge compared with those before operation. General anesthesia does not aggravate the severity of encephalopathy, and endoscopic treatment under general anesthesia with tracheal intubation is effective for HE patients complicated with AVB.

## 1. Introduction

Liver cirrhosis is a widely prevalent chronic progressive liver disease, and is associated with high morbidity and mortality, which accounts for approximately 1 million deaths worldwide annually.^[1]^ Undoubtedly, the high mortality is mainly attributed to its related complications. Esophagogastric variceal bleeding is one of the most common complications and it is often life-threatening. At present, the treatment methods for rupture and bleeding of gastroesophageal varices include drug therapy, compression hemostasis, endoscopic therapy, and interventional and surgical operations. Endoscopy and intervention or their combined application is the mainstream treatment modes in clinical practice.^[2]^ Active variceal bleeding (AVB) is a medical emergency that requires swift intervention to stop the bleeding and achieve durable hemostasis. The bleeding time is one of the important factors that affecting the prognosis of patients with massive bleeding. Therefore, timely and effective treatment of the acute bleeding episode can improve the survival.^[3]^ When vasoactive drugs and volumetric resuscitation are not effective in controlling AVB, early endoscopic therapy is an option for patients at high-risk of further bleeding or death to improve the survival of cirrhotic patients with AVB.^[4,5]^

Hepatic encephalopathy (HE) is a serious and potentially fatal complication of liver cirrhosis, refers to a clinical syndrome of reversible neuropsychiatric impairment of varying severity. The majority of patients with cirrhosis who present with an episode of overt HE will have an underlying precipitating factor and management aimed at correcting the underlying cause will usually reverse encephalopathy.^[6]^ Gastrointestinal bleeding is a common precipitant that induces the occurrence of HE. In the circumstance of HE complicated with AVB, controlling the variceal bleeding is of great importance to achieve hemodynamic stabilization and the recovery of consciousness disturbance. However, when pharmacological treatments aim at reducing the risk of bleeding from varices has no effect to stop bleeding while transjugular intrahepatic portosystemic shunt (TIPS) is limited in HE, the endoscopic treatment is an alternative to save the life of patient.^[7]^

Emergency endoscopy for AVB requires careful airway protection and the patient cooperation. Thus, the application of general anesthesia with tracheal intubation is a better way in the process of endoscopic operations.^[8]^ Actually, anesthesia complications among endoscopic procedures in cirrhosis are rare.^[9]^ The challenge for anesthesia in cirrhotic patient with encephalopathy is that narcotics and sedatives may aggravate the degree of consciousness disturbance. Previous study found that sedative such as propofol for upper gastrointestinal endoscopy does not exacerbate minimal hepatic encephalopathy in patients with cirrhosis.^[10,11]^ However, few reports provide evidence concerning the feasibility of general anesthesia in HE patients with AVB. Due to the scarcity of this condition, we analyzed 36 cirrhotic patients complicated with encephalopathy and AVB who received or not, the endoscopic therapy under the general anesthesia with tracheal intubation, aim to study the feasibility, safety and short-term efficacy of this clinical practice.

## 2. Patients and methods

### 2.1. Design, population and sample

A retrospective analysis of all relevant patient records at the first Affiliated Hospital of Chengdu Medical College (Chengdu, China) and the Affiliated Hospital of Southwest Medical University (Luzhou, China) was performed for the study period from January 1, 2017, to June 30, 2022. All patients included in this study were diagnosed HE with AVB. There were 14 patients (11 males and 3 females) included in the anesthesia group from an initial pool of 18 patients, while 22 patients in the control group. The anesthesia group was treated with endoscopic operation under general anesthesia with tracheal intubation since the conventional pharmacological treatment had no obvious effect on variceal bleeding. Patients in the control group received pharmacological treatment, or and early endoscopic therapy without general anesthesia. According to the principles of the “Declaration of Helsinki,” this study was reviewed and approved by the Ethics Committee of the First Affiliated Hospital of Chengdu Medical College. As this was a retrospective study of de-identified data, it did not involve patient consent.

Patients confirmed to have HE required features as outlined in the 2014 revision of the AASLD/EASL Practice Guidelines^[12]^ and had elevated blood ammonia level. The stage of clinical HE was classified according to the 2014 Practice Guideline by AASLD/EASL^[12]^ at the time of admission. Variceal bleeding is defined as bleeding from a varix at the time of endoscopy. Varices are accepted as the bleeding source, when a venous spurt is seen or when there is fresh bleeding in the presence of varices. In the absence of active bleeding, either a “white nipple sign,” adherent clots on varices or the presence of medium or large varices with no other potential bleeding lesions suggest varices as the source of hemorrhage.

Patients with acute cerebral infarction, acute myocardial infarction, alcohol withdrawal syndrome, psychiatric diseases, drug overdose, electrolyte disturbances were excluded. In addition, patient had received endoscopic treatment but failed to stop bleeding was also excluded in this study.

### 2.2. Anesthesia procedure and data collection

Anesthesia was induced by 0.8 mg/kg atracurium, 1.5 mg/kg propofol and 0.4 μg/kg sufentanil. After tracheal intubation, anesthesia was maintained with 2% to 3% sevoflurane and continuous pumping 0.1 μg·kg^-1^. min^-1^ remifentanil.

Data were gathered from the patients admission charts, Hospital physical and digital archives on admission and at discharge. Age, sex, etiology of liver cirrhosis, History of TIPS, and splenectomy, diabetes, portal vein thrombosis, blood level of alanine transaminase (ALT), aspartate aminotransferase (AST), albumin, total bilirubin (TB), ammonia, prothrombin time (PT), the type of endoscopic surgery, stage of HE, and hospital stay was recorded. We calculated Child-Pugh score and Child-Pugh class according to medical records.

## 3. Statistical analysis

Statistical analysis was performed using SPSS version 25.0 (IBM SPSS Inc, Chicago, Illinois). Continuous data are expressed as medians with interquartile ranges [M (P25–P75)]. For abnormally distributed variables and ranked data, the Mann–Whitney *U* test was used for comparisons. Categorical data are presented as numbers and Percentages and were compared by the chi-square test or Fisher exact test. *P* < .05 was considered to be statistically significant.

## 4. Results

### 4.1. Patient characteristics

In total, 36 cirrhotic patients complicated with encephalopathy and AVB were included in this study. There were 28 (77.8 %) male patients and 8 (22.2 %) female patients, and the ratio of male versus female was 3.5:1. The age range of the patients admitted in this study was from 34 to 76 years. Chronic hepatitis B (50.0 %) was the predominant etiology for liver cirrhosis in our study population, followed by alcohol (38.9 %) was the second cause. One patient had the etiology of alcohol and hepatitis C, and 1 patient had alcohol and primary biliary cholangitis. HE is a common complication of TIPS, there were 2 (5 %) patients had ever received TIPS. Table 1 summarizes the characteristics of this study population.

**Table 1:** Baseline characteristics of patients on admission.

Characteristics	
Age, yr	56.97 (34–76)
Male/females, n	28/8
Etiology, n (%)	
HBV	18 (50%)
HCV	1 (3%)
Alcohol	14 (38.9%)
PBC	1 (3%)
Other	3 (8%)
HCC advanced, n	2
PVT, n	1
History of TIPS, n	2
History of splenectomy, n	3
Diabetes, n	5
Child-Pugh score (mean ± SD)	11.58 ± 2.26
Child-Pugh class, n (%)	
A	1 (3%)
B	5 (13.9%)
C	30 (83.3%)
Stage of HE n (%)	
I	12 (33.3 %)
II	10 (27.8 %)
III	7 (19.4 %)
IV	7 (19.4 %)

Data were mean ± standard deviation, or numbers and percentages, as appropriate.

HBV = hepatitis B virus, HCC = hepatocellular carcinoma, HCV = hepatitis C virus, HE = hepatic encephalopathy, n = number, PBC = primary biliary cholangitis, PVT = portal vein thrombosis, TIPS = transjugular intrahepatic portosystemic shunt.

The average of Child-Pugh score in this study is 11.58 ± 2.26, and the grade was mostly Child-Pugh class C (83.3%) on admission, only 1 patient was graded Child-Pugh class A (3%). The stage of HE of I, II, III, IV on admission was 12 (33.3 %), 10 (27.8 %),7 (19.4 %),7 (19.4 %) respectively.

### 4.2. General anesthesia did not aggravate the severity of HE

The patients’ characteristics on admission was evaluated between the anesthesia group and control group. No significant difference of the blood level of ALT, AST, albumin, TB, PT was observed between the 2 groups, the same was investigated in Child-Pugh score, Child-Pugh class and stage of HE (Table 2). We next compared the characteristics at discharge between patients in the anesthesia group and control group to evaluate whether anesthesia aggravated the liver function and conscious disturbance of encephalopathy. As table 3 showed, except albumin, there were no statistically significant differences between the 2 groups in terms of ALT, AST, TB, PT, and Child-Pugh score, Child-Pugh class. Furthermore, the stage of HE was all improved in the 2 groups, 12 (85.8 %) patients were totally recovered from consciousness disturbance in the anesthesia group and 16 (72.7 %) in the control group respectively, the difference between the 2 groups was not significant (*P*>0.05). Except the death cases, there were still 3 patients who had impaired consciousness at discharge in the control group.

**Table 2:** The patients backgrounds and nonparametric tests in patients of two groups on admission.

	Anesthesia group (n = 14)	Control group (n = 22)	*P* value
Age, yr	56.86 (34–76)	57.05 (37–74)	.961
Gender			1 (fisher)
Males	11	17	
Females	3	5	
Etiology n (%)			.079
HBV	4 (28.6%)	12 (54.5%)	
HCV	0	2 (9.1%)	
Alcohol	9 (64.3%)	5 (22.7%)	
Others	1 (7.1%)	3 (13.6%)	
HCC, n	1	1	1 (fisher)
PVT, n	0	1	1 (fisher)
History of TIPS, n	0	3	.267 (fisher)
History of splenectomy, n	1	2	1 (fisher)
ALT, U/L	29.5 (24–40.5)	32.85 (21.6–49.78)	.746
AST, U/L	44.5 (35.25–82.75)	45.3 (31.5–94.75)	.948
Albumin, g/L	28.8 (23.63–31.5)	25.15 (22.15–29.3)	.256
TB, μmmol/L	40.4 (19.85–60.88)	60.95 (26.6–109.83)	.23
Prothrombin time, sec	19.1 (17.85–21.33)	21.35 (17.9–23.58)	.355
Ammonia, μmmol/L	109.6 (92.28–133.15)	97.3 (82.05–129.18)	.256
Child-Pugh score (mean ± SD)	10.71 ± 2.12	12.14 ± 2.11	.922
Child-Pugh class, n (%)			.441
A	1 (7.1%)	0	
B	2 (14.3%)	3 (13.6%)	
C	11 (78.6%)	19 (86.4%)	
Stage of HE n (%)			.109
I	6 (42.9%)	6 (27.3%)	
II	6 (42.9%)	4 (18.2%)	
III	1 (7.1%)	6 (27.3%)	
IV	1 (7.1%)	4 (18.2%)	

Data were mean ± standard deviation, or numbers and percentages, or median (25th–75th percentile), as appropriate.

ALT = alanine transaminase, AST = aspartate aminotransferase, HBV = hepatitis B virus, HCC = hepatocellular carcinoma, HCV = hepatitis C virus, HE = hepatic encephalopathy; n, number, PVT = portal vein thrombosis, TB = total bilirubin, TIPS = transjugular intrahepatic portosystemic shunt.

**Table 3:** The nonparametric tests in patients of two groups at discharge.

	Anesthesia group (n = 14)	Control group (n = 22)	*P* value
ALT, U/L	23.5 (18–49)	29.7 (18.53–54.48)	.697
AST, U/L	41.5 (26.25–59.75)	51 (33.63–82.22)	.455
Albumin, g/L	32.95 (30.75–34.6)	27.65 (23.22–31.28)	.007
TB, μmmol/L	41.8 (24.13–57.9)	52.8 (23.5–81.3)	.592
Prothrombin time, sec	17.9 (16.08–20.85)	19.9 (17.38–24.85)	.211
Ammonia, μmmol/L	52.75 (41.48–79.7)	46.1 (40.58–65.75)	.496
Child-Pugh score (mean ± SD)	8.85 ± 2.76	10.32 ± 2.95	.781
Child-Pugh class, n (%)			.366 (fisher)
A	3 (21.4%)	2 (9.1%)	
B	6 (42.9%)	8 (36.4%)	
C	5 (35.7%)	12 (54.5 %)	
Stage of HE n (%)			.887 (fisher)
Non of HE	12 (85.7%)	16 (72.7%)	
0	0	0	
I	0	1 (4.5%)	
II	0	0	
III	0	2 (9.1%)	
IV	2 (14.3%)	3 (13.6%)	
Hospital stays	9.5 (7.5–12.5)	8.5 (3.25–16.5)	.782

Bold italic indicates significant *P* value. Data were mean ± standard deviation, or numbers and percentages, or median (25th–75th percentile), as appropriate.

ALT = alanine transaminase, AST = aspartate aminotransferase, TB = total bilirubin, n = number, HE = hepatic encephalopathy.

### 4.3. Endoscopic treatment under tracheal intubation anesthesia was helpful to HE patients with AVB

**Figure 1.**
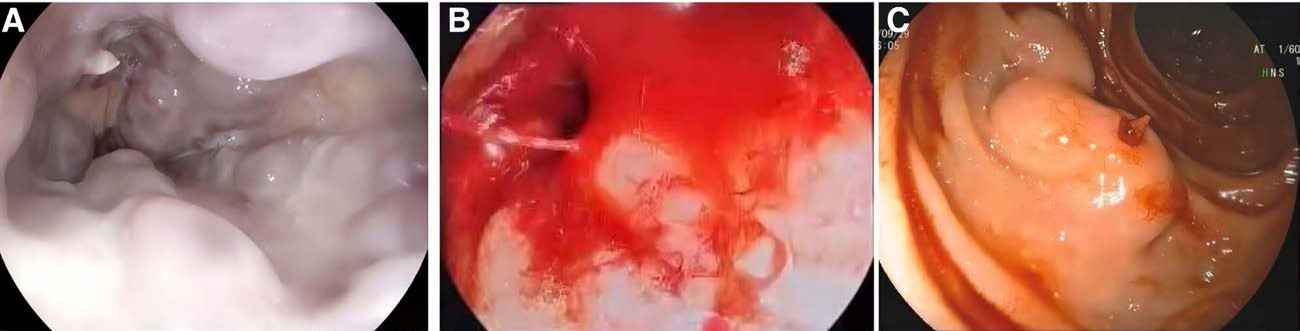
Presentation of variceal bleeding under endoscopic examination. (A) white clot on the esophageal varices. Variceal venous spurting in esophageal varices (B) and duodenal varices (C).

As shown in Figure 1, variceal venous spurt, adhesion of white clot could be investigated in present study. All patients in the anesthesia group received endoscopic treatment under tracheal intubation general anesthesia when the conventional pharmacological treatment had no obvious effect on variceal bleeding. Whereas there are only 5 patients in the control group received endoscopic therapy under local anesthesia due to the attending physician’s concern about potential adverse effects of anesthetic drugs, among them, 4 patients were given endoscopic variceal ligation combined with gastric tissue glue injection, and 1 received tissue glue injection to duodenal varices.

**Figure 2.**
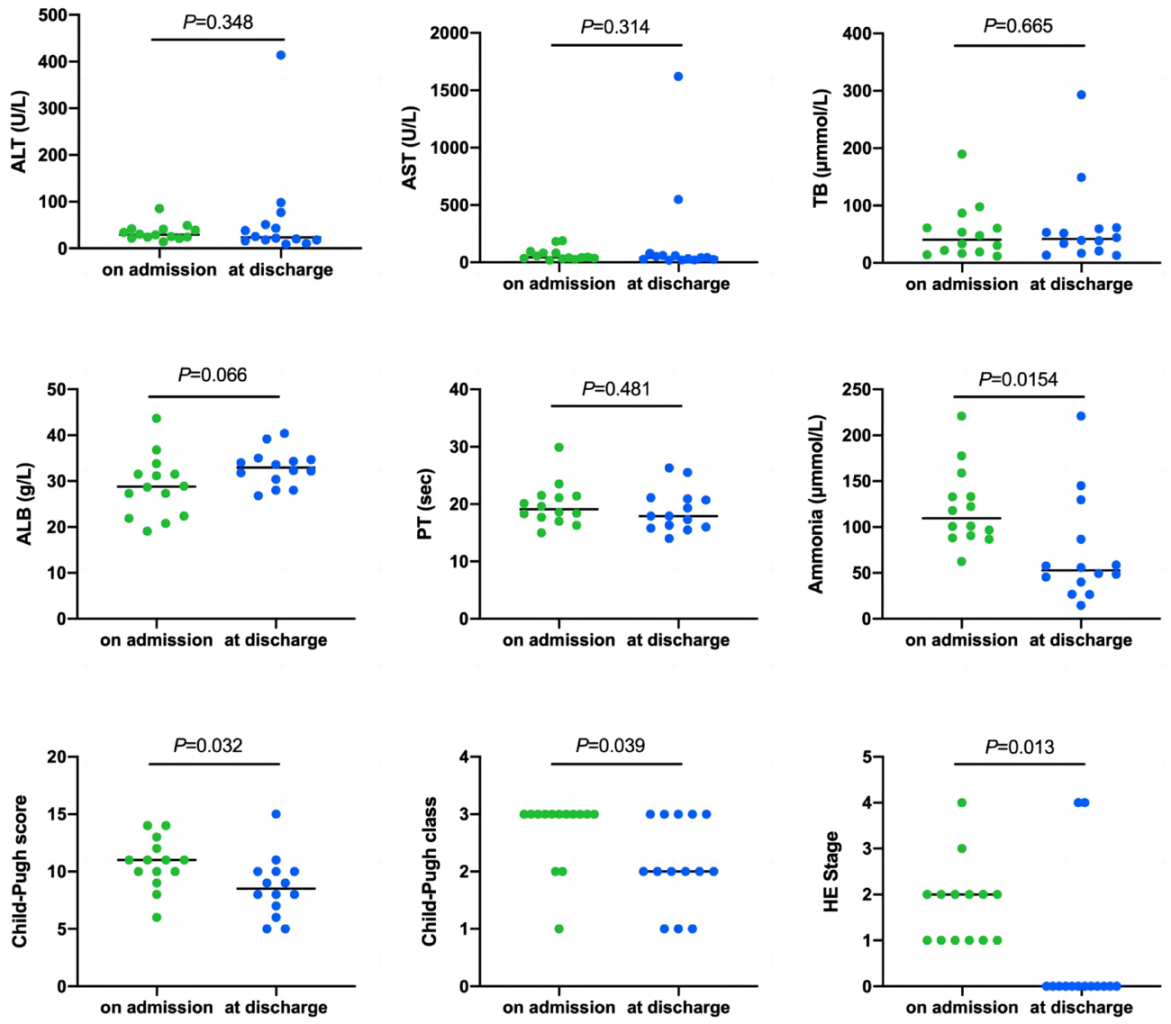
Child-Pugh score, Child-Pugh class and the stage of HE was improved significantly compared with that of pre-operation in the anesthesia group (*P* < .05). No significant difference was observed in the blood level of ALT, AST, TB, ALB, and PT. ALT = alanine transaminase. AST = aspartate aminotransferase, ALB = albumin, TB = total bilirubin, PT = prothrombin time, HE = hepatic encephalopathy.

We further investigated the hepatic function and severity of encephalopathy of patients in the anesthesia group to evaluate the potential effect of general anesthesia with tracheal intubation to HE patients with AVB. The results showed the blood level of ammonia, Child-Pugh score, Child-Pugh class and the stage of HE at discharge were significantly improved compared with that of pre-operation (*P* < .05), while no significant difference was observed in the blood level of ALT, AST, TB, albumin and PT (Fig. 2).

### 4.4. The characteristics of death cases

We tried to analyze the risk factor of death in present study by using logistics regression method, but the number of cases is not sufficient for this analysis. Therefore, we listed the related information of death cases (Table 4). The total mortality rate in this series was 13.9% (5/36), 2 (14.3%) in the anesthesia group, 3 (13.6%) in the control group, all were male patients, and the median age of death was 52 years old (range, 34–71 years). The child-Pugh class of death cases were all grade C. 3 (60%) of them were in the stage IV of HE, accounted for 42.9% of all the patients with stage IV included in the study. The death cases in the control group were all treated with pharmacological therapy and volume resuscitation without any endoscopic treatment or TIPS.

**Table 4:** The characteristics of death cases.

Characteristics	Case 1	Case 2	Case 3	Case 4	Case 5
Age (yr)	34	55	56	52	71
Gender	Male	Male	Male	Male	Male
Etiology	Alcohol	Alcohol	Alcohol	HBV	Alcohol
Child-Pugh score	11	14	14	15	12
Child-Pugh class	C	C	C	C	C
Stage of HE	II	IV	I	IV	IV

HE = hepatic encephalopathy, HBV = hepatitis B virus.

## 5. Discussion

The present study provides new information of general anesthesia in cirrhotic patients with encephalopathy and AVB undergoing endoscopic treatment. Specifically, we found that general anesthesia did not aggravate the degree of encephalopathy, and endoscopic treatment under tracheal intubation anesthesia was safe and effective in these patients. The Child-Pugh score, Child-Pugh class and the stage of HE improved greatly after receiving endoscopic surgery with tracheal intubation anesthesia. Furthermore, there were not significantly differences in liver function and stage of HE between patients who received and did not receive general anesthesia. In addition, our study demonstrates that Child-Pugh class C and stage IV of HE seems to associate with worse clinical outcomes in cirrhotic patients complicated with encephalopathy and AVB.

HE is a significant complication of severe acute or chronic liver insufficiency that is characterized predominantly by alterations of personality, consciousness, cognition and motor function ranging from subclinical alterations to coma.^[6]^ As one of the serious complications of liver cirrhosis, HE contributes greatly to impaired quality of life, high rates of morbidity and mortality in cirrhotic patient. In addition to ammonia-lowering strategies, identification and correction of precipitating factors, such as infection, gastrointestinal bleeding, constipation, electrolyte disturbances, sedatives should also be managed in the treatment of HE. Therefore, for HE patients with variceal bleeding, it is of great importance to control gastrointestinal hemorrhage effectively. Apart from HE, acute gastrointestinal variceal bleeding per se is an immediate life-threatening condition and a major cause of significant morbidity and mortality in patients with cirrhosis.^[13]^ The early mortality rate of AVB in cirrhotic patients ranges from 7% to 14.6%, and the mortality rate from rebleeding is also high.^[4,14,15]^ Therefore, prompt and appropriate management is important in patients with AVB.^[16]^ Early endoscopy (within 12 hours) is recommended in patients with AVB who were at high-risk for further bleeding or death, and thus can improve the survival of cirrhotic patients.^[4,17,18]^ Previous reports demonstrate that delayed endoscopy seems to be associated with higher rebleeding and mortality in patients with hematemesis and AVB.^[17,19]^ TIPS has been considered as a salvage treatment in patients with severe variceal bleeding who failed control of bleeding,^[5]^ but there is a relative contraindication in patients with encephalopathy due to an increased risk of morbidity and complications that relate to early mortality.

Endoscopic therapies are often used in combination with pharmacological therapy in preventing and controlling variceal bleeding. Utilization of anesthesia service in endoscopic operations can facilitate the procedural conditions and tracheal intubation may benefit patients with upper gastrointestinal bleeding for airway protection. In patients with altered consciousness, endoscopy should be performed with protection of the airway.^[20]^ With the help of anesthesiologists managing the airway and hemodynamics for patients, endoscopists can focus more on endoscopic operation.^[8]^ However, the application of narcotics and sedatives in patients with HE is a significant challenge and availability of this procedure is often limited. For these reasons, we assessed the safety and therapeutic efficacy of endoscopic treatment under general anesthesia with tracheal intubation in HE patients with AVB.

We studied 36 patients in present study, endoscopic therapy was performed in all patients in the anesthesia group, while only 5 patients in the control group received endoscopic treatment but did not receive general anesthesia. There are no universally agreed suggestions exists to the application of general anesthesia and the airway management required in patients with AVB undergo endoscopic procedure. However, results in present study showed the Child-Pugh score, Child-Pugh class and the stage of HE was all improved significantly in both groups. The Child-Pugh score, Child-Pugh class and the stage of HE in the anesthesia group were significantly improved at discharge compared with those before operation. Apparently, endoscopic treatment under general anesthesia had therapeutic efficacy to stop variceal bleeding, and general anesthesia did not adversely impact HE patient survival or clinical outcomes. Endoscopic therapy is effective in controlling AVB especially in Child-Pugh class C cirrhotic patients while early TIPS has not been confirmed the effect on survival of these patients.^[21,22]^ Child-Pugh was superior to the other factors in HE patients for prediction of in-hospital mortality^[23,24]^, and also an important risk factors for predicting postoperative mortality in cirrhotic patients.^[25]^ Five death cases in present study were all Child-Pugh class C, which is consistent with previous research. Furthermore, the other point to note is that the majority (60%) of the deaths were in stage IV of HE, and higher stage of HE seems to correlate with a worse prognosis.^[26]^

The anesthesia management of patients with cirrhosis has its particularity, which involves the comprehensive consideration of narcotic drug metabolism disorder, hyperdynamic circulation, perioperative hypoxemia, coagulation dysfunction, thrombosis and hepatic encephalopathy.^[27,28]^ Anesthesia in chronic liver disease is a pretty challenging condition for every anesthesiologist, this potential adverse effect could be diminished by meticulous attention on optimizing the patient’s condition preoperatively and choosing appropriate anesthetic regimen and drugs in this setting. Drug choice and dose adjustments should be more considered for patients with advanced disease as evidenced by encephalopathy. Sedatives such as midazolam and diazepam, which were avoided in the present study in the context of encephalopathy. The drugs used during anesthesia in our study are known to have minimal effects on liver function. In present study, propofol served as a good alternative for sedation and induction of anesthesia, as it had minimal effects on the cognitive function in patients with minimal hepatic encephalopathy.^[29]^ Degradation of atracurium by Hofmann elimination and ester hydrolysis depends mainly on pH and temperature and is independent of liver and kidney function.^[30]^ Narcotics like sufentanil and in conjunction with some volatile anesthetics like sevoflurane or intravenous anesthetics like propofol are recommended in cirrhotic patients.^[24,31,32]^ Remifentanil is the safest agent as it is metabolized by the red cell esterase, rather than the hepatocytes.^[33]^ Previous study tried to use remifentanil in a patient with hepatic failure and mild encephalopathy to provide intraoperative and postoperative analgesia, and concluded that remifentanil can provide perioperative analgesia in patients at risk of developing HE.^[34]^ In present study, remifentanil was used in the maintenance of anesthesia in the anesthesia group since that it has a short duration of action and does not require hepatic metabolism. However, in cirrhotic patients with HE complicated with acute variceal hemorrhage, it remains to be further explored whether the judicious choosing of appropriate anesthetic drugs, tailored anesthetic monitoring and management, and airway protection have a beneficial effect on the mortality of patients, but it does not seem to bring additional adverse outcomes to this specific group of patients.

There are several study limitations to consider as well. The most important limitation is the small number of patients analyzed although we collected cases in 2 regional central hospital. A portion of HE patient with upper gastrointestinal bleeding did not undergo endoscopic examination during this hospitalization and these patients were excluded in this group. The other fact is that very few clinicians would perform endoscopic treatment under general anesthesia when cirrhotic patients complicated with encephalopathy. The small sample size limits the statistical power and feasibility of the results and makes the results prone to biases. A second limitation of this study is due to its retrospective nature and all data were collected retrospectively. Additionally, we depended on evaluation of available clinical and imaging data and the time of data collection may not be consistent.

## 6. Conclusion

To our knowledge, this study is the first study focused on the possible effect of general anesthesia on cirrhotic patients complicated with encephalopathy and AVB. Our results showed endoscopic treatment under general anesthesia with tracheal intubation did not exacerbate the severity of encephalopathy and had a therapeutic effect to HE patients with AVB. However, further prospective evaluation will be needed to determine whether general anesthesia with appropriate anesthetic drugs could be a safe anesthesia in cirrhotic patient with encephalopathy, and endoscopic treatment under general anesthesia with tracheal intubation could benefit these patients with AVB.

## Author contributions

**Data curation:** Lin Wen, Rui Zhong.

**Formal analysis:** Xia Chen.

**Investigation:** Lin Wen.

**Methodology:** Tao Chen.

**Software:** Rui Zhong.

**Supervision:** Xia Chen

**Validation:** Tao Chen.

**Writing – original draft:** Tao Chen.

**Writing – review & editing:** Xia Chen.
